# Pesticide Spraying and Health Effects

**DOI:** 10.1289/ehp.113-a150a

**Published:** 2005-03

**Authors:** Grace Ziem

**Affiliations:** Occupational and Environmental Health, Emmitsburg, Maryland, Telephone: (301) 241-4346, Fax: (301) 241-4348

I noticed with interest the article “Pesticide Spraying for West Nile Virus Control and Emergency Department Asthma Visits in New York City, 2000” by Karpati et al. (2004). I am a physician who treats hundreds of patients with chronic illness from chemical overexposure. Many of these patients have toxic encephalopathy, reactive airway disease, and other chemically induced organ system damage. When my patients become ill from pesticide spraying, they usually do not head for an emergency room, where they typically experience long waits in an environment containing germicidal residue, scented products, carbonless copy paper, hospital linens with heavy fabric softener, and other exposures. In addition, they have learned from experience that emergency department personnel often do not understand their condition and do not know how to treat it. Thus your survey, while with admirable intent, greatly underestimates the problem of respiratory exacerbation from West Nile virus pesticide use. Many of my patients have experienced severe neurologic and respiratory exacerbations as well as other organ system damage, such as significant increase in liver enzymes, from exposure to residue from pesticide spraying for West Nile virus. In addition, it is my understanding that these pesticides are not effective for controlling adult mosquitoes and that the Centers for Disease Control and Prevention and other authorities recommend larvae control. The extent of exacerbation of illness caused by pesticide use for West Nile virus control is likely greater than the number of cases of West Nile virus.

Persons who are at increased risk for symptom exacerbation from pesticide spraying such as that used for West Nile virus control include individuals with migraines, chronic sinus problems, asthma, reactive airway disease, autoimmune diseases (many of which are exacerbated by pesticide exposure), and conventional allergies (Kipen et al. 1994). There is increased respiratory inflammation with conventional allergies, and pesticides more readily enter the body because the barrier function of the respiratory tract is further compromised. In addition, Karpati et al. (2004) failed to take note of the U.S. Environmental Protection Agency (EPA) final report “Principles of Neurotoxicity Risk Assessment” (U.S. EPA 1994). This document confirmed the lack of a blood–brain barrier between the nose and the brain, so that pesticides readily enter the body through the nose and pass directly to the brain. This report further confirmed the unusual vulnerability of the brain to neurotoxicants: pesticides are lipophilic and therefore seek out lipid tissue such as the brain, and because the brain has unusually long neurons, repair of damage in the neurons occurs much less readily than in other body cells.

Other groups at increased risk of pesticides are those with chronic obstructive lung disease, toxic encephalopathy, and neural degenerative diseases. Pyrethroid pesticides are significant neurotoxins (Eells et al. 1992; McDaniel and Moser 1993; Tippe 1993; Vijverberg and van den Bercken 1990), and because they are increasingly replacing organophosphates, they now account for a large proportion of the pesticide-induced chronic illness among my patients. Emergency room visits are merely the tip of the iceberg, and patients with many of these disorders usually avoid the emergency room. Thus, the use of emergency rooms is not a sensitive indicator of body damage from pesticides.

In my experience, the use of nebulized glutathione, the major antioxidant and major detoxifying agent of the body (Klaassen et al. 1986), when combined with lipoic acid, helps to improve an individual’s ability to detoxify (Packer et al. 1995); lipoic acid reactivates glutathione in lipid-and water-based tissues. Also, nebulized glutathione combined with adequate buffered vitamin C reactivates glutathione in water-based tissues.
